# Derivatization‐free determination of short‐chain volatile amines in human plasma and urine by headspace gas chromatography‐mass spectrometry

**DOI:** 10.1002/jcla.23062

**Published:** 2019-10-08

**Authors:** Peter Neyer, Luca Bernasconi, Jens A. Fuchs, Martina D. Allenspach, Christian Steuer

**Affiliations:** ^1^ Institute of Laboratory Medicine Kantonsspital Aarau Aarau Switzerland; ^2^ Institute of Pharmaceutical Sciences ETH Zurich Zurich Switzerland

**Keywords:** derivatization‐free multi‐analyte procedure, headspace‐GC‐MS, method validation, volatile amines

## Abstract

**Background:**

Short‐chain volatile amines (SCVA) are an interesting compound class playing crucial roles in physiological and toxicological human settings. Dimethylamine (DMA), trimethylamine (TMA), diethylamine (DEA), and triethylamine (TEA) were investigated in detail.

**Methods:**

Headspace gas chromatography coupled to mass spectrometry (HS‐GC‐MS) was used for the simultaneous qualitative and quantitative determination of four SCVA in different human body fluids. Four hundred microliters of Li‐heparin plasma and urine were analyzed after liberation of volatile amines under heated conditions in an aqueous alkaline and saline environment. Target analytes were separated on a volatile amine column and detected on a Thermo DSQ II mass spectrometer scheduled in single ion monitoring mode.

**Results:**

Chromatographic separation of selected SCVA was done within 7.5 minutes. The method was developed and validated with respect to accuracy, precision, recovery and stability. Accuracy and precision criteria were below 12% for all target analytes at low and high levels. The selected extraction procedure provided recoveries of more than 92% from both matrices for TMA, DEA and TEA. The recovery of DMA from Li‐heparin plasma was lower but still in the acceptable range (>75%). The newly validated method was successfully applied to plasma and urine samples from healthy volunteers. Detected concentrations of endogenous metabolites DMA and TMA are comparable to already known reference ranges.

**Conclusion:**

Herein, we describe the successful development and validation of a reliable and broadly applicable HS‐GC‐MS procedure for the simultaneous and quantitative determination of SCVA in human plasma and urine without relying on derivatization chemistry.

## 1 INTRODUCTION

Dimethylamine (DMA), trimethylamine (TMA), diethylamine (DEA) and triethylamine (TEA) are short‐chain aliphatic amines (SCVA) and used as biomarkers for the identification of different physiological, pathophysiological and toxicological states in human. DMA and TMA are present in several human body fluids like urine, blood and sweat.^1^ Bacterial metabolism results in the transformation of phosphocholine (PC) and choline—derived from meat, seafood, and dairy products—to TMA.^2^, ^3^, ^4^ In recent years, the non‐odorous and non‐volatile oxidation product of TMA—trimethylamine‐N‐oxide (TMAO)—attracted the attention of physicians and clinical chemists worldwide.^5^, ^6^, ^7^, ^8^ TMAO is suspected to be of diagnostic or prognostic value, respectively, in cardio vascular events and pneumological diseases like community acquired pneumonia.^9^, ^10^ Oxidation of TMA to TMAO is catalyzed by flavin‐monooxygenases (FMO) in liver microsomes.^11^ Genetic mutations and liver damage can lead to a loss in FMO activity and result in trimethylaminuria, also known as fish odor syndrome. Patients suffering from this condition are characterized by penetrant smell of fish resulting from the transpiration and expiration of excess TMA. Until now, no cure from this disease is reported and the only way to ameliorate the symptoms is to avoid choline‐rich nutrition.^12^ DMA is present in a variety of nutrients. An increase of the urinary DMA concentration can be observed, for example, after the consumption of seafood.^13^ In humans, degradation of glycine and sarcosine and subsequent methylation of monomethylamine lead to DMA formation. Additionally, DMA is a metabolite of asymmetric dimethyl arginine (ADMA). Asymmetric dimethyl arginine is known as inhibitor of nitrogen oxide synthase, playing a significant role in several pathophysiological states like renal diseases and chronic obstructive pulmonary disease.^14^, ^15^ DEA and TEA are closely related short‐chain amines which are not derived metabolically. However, DEA and TEA are widely distributed reagents in pharmaceutical and chemical industries.^16^ TEA irritates human mucous membranes and causes headache and nausea.^17^ Nitrosation of DMA and DEA results in the formation of N‐dimethyl‐nitrosamine (NDMA) and N‐diethyl‐nitrosamine (NDEA), respectively. Both compounds are classified as cancerogens.^18^, ^19^, ^20^ NDMA and NDEA are currently found as impurities in several active pharmaceutical ingredients (eg, Valsartan and Irbesartan) and perturb national authorities.^21^, ^22^, ^23^ Formation of nitrosamine is also reported in human and animal gastric juice and is accelerated by bacteria in urinary tract infections.^18^, ^24^

SCVA were mostly detected by gas chromatography (GC) after direct injection.^1^ Additionally, determination of TMA is also performed by liquid chromatography.^25^ However, in food and bioanalysis, headspace (HS)‐GC is preferred due to (a) the low contamination of the GC column and injection system and (b) to the high sensitivity and repeatability. In the context of SCVA determination, different derivatization procedures were described for quantification.^26^, ^27^ Although chemical derivatization of target analytes is a common preanalytical procedure, this additional step is often time‐consuming and causes an additional source of error. In combination with HS‐GC, dynamic extraction techniques—like solid‐phase microextraction (SPME)—are often reported to further improve detection limits of SCVA. However, SPME needs a higher grade of equipment.^28^

In here, we report a derivatization‐free and static HS‐GC method using a volatile amine column for human plasma and urine samples, respectively. To the best of our knowledge, no HS‐GC‐MS assay for the simultaneous analysis of the selected SCVA has been reported in human body fluids. The method was validated according to international guidelines^29^ and can easily be applied even in less equipped laboratories offering short preparation and analysis times.

## 2 EXPERIMENTAL

### 2.1 Materials

Analytical reference standard of TMA was obtained from Sigma‐Aldrich. Dimethylamine (2 mol/L solution in methanol), DEA, TEA, isopropylamine, carnitine, and choline were also purchased from Sigma‐Aldrich and were of highest analytical grade. Deuterated TMA_d9_ was obtained from Toronto Research Chemicals. Sodium hydroxide (NaOH), hydrochloric acid solution (HCl), and potassium chloride (KCl) were purchased from VWR. Phosphate‐buffered saline (PBS) was from Gibco Life technologies. Headspace vials, aluminum caps, and MS‐septa were purchased from Infochroma. Pure water was generated from an in‐house water purification system from Labtec. For all experiments, Gilson pipettes and Gilson DIAMOND tips were used. Lithium‐heparin tubes without gel separator were from BD (Becton Dickinson).

### 2.2 Apparatus

All samples were analyzed using a Focus Trace GC Ultra with Triplus Headspace injection system and DSQ II MS detector (Thermo Scientific). Chromatographic separation was performed on a Restek Rtx‐Volatile Amine column (30 m; 0.32 mm ID; 5 µm; BGB). Helium flow was set to constant flow at 2 mL/min. Split ratio was set to 7. The starting temperature for the oven was 40°C and held for 4 minutes. Temperature was increased with 25°C/min to 250°C and kept constant for further 3 minutes. Headspace conditions were as follows: agitating for 10 minutes at 70°C. Syringe temperature was set to 80°C and 2 mL of gaseous sample were drawn from the headspace and injected. Injector and transfer line to the MS detector were set to 200°C and 230°C, respectively. MS was performed in positive electron impact (EI) mode at 70 eV, and the temperature of the EI source was set to 200°C. The MS was operated in single ion monitoring mode (SIM m/z, DMA: 44; TMA 44, 59; IPA 44, 59; DEA: 44, 73; TEA 73, 86, 101).

### 2.3 Preparation of calibration and QC samples

Separate stock solutions for multi‐analyte calibration (Cal) and quality control (QC) samples were prepared in 0.1 mol/L HCl. All solutions were stored in aliquots at −20°C. The final calibration concentrations are given in Table 1 for each target analyte. Isopropylamine (IPA) was used as internal standard (100 µmol/L in 0.1 mol/L HCl).

**Table 1:** Method validation data: retention time (R_t_) and analytes concentration for Cal and QC used for method validation are written in bold. Mean back‐calculated concentration according to regression equations (n = 6) are shown in italic

Analyte	R_t_ (min)	Cal 1 [µmol/L]	Cal 2 [µmol/L]	Cal 3 [µmol/L]	Cal 4 [µmol/L]	Cal 5 [µmol/L]	Cal 6 [µmol/L]	QC high [µmol/L]			QC Med [µmol/L]									QC low [µmol/L]		
								Bias (%)	RSD_T_ (%)	RSD_R_ (%)	Bias (%)	RSD_T_ (%)	RSD_R_ (%)	RE (RSD)	( %)	Plasma	RE (RSD)	(%)	Urine	Bias (%)	RSD_T_ (%)	RSD_R_ (%)
DMA	2.3	12 500	8000	4000	400	50	25	10 000			1000			75.4 (5.1)			112.5 (5.2)			33.3		
		*12 093.5*	*8202.6*	*4370.5*	*816.8*	*137.1*	*23.1*	*−4.0*	*5.3*	*5.0*	*−1.5*	*2.3*	*3.8*							*−6.6*	*11.7*	*9.6*
TMA	2.56	500	320	160	16	2.0	1.0	400			40			92.6 (7.0)			93.0 (3.4)			1.3		
		*498.2*	*319.0*	*163.3*	*32.5*	*6.2*	*0.9*	*0.2*	*6.8*	*6.0*	*−1.6*	*1.2*	*6.8*							*−3.6*	*2.1*	*4.9*
IPA (IS)	3.27																					
DEA	5.47	5000	3200	1600	160	20	(10)	4000			400			97.2 (4.5)			99.0 (3.4)			13.3		
		*4806.4*	*3321.7*	*1766.0*	*329.6*	*51.4*	*9.3*	*−6.8*	*6.4*	*5.4*	*−6.9*	*1.8*	*6.0*							*−11.5*	*12.6*	*12.6*
TEA	7.35	250	160	80	8.0	1.0	0.5	200			20			98.1 (6.4)			104 (3.6)			0.67		
		*243.1*	*163.7*	*86.2*	*16.3*	*2.8*	*0.5*	*3.8*	*6.0*	*5.9*	*5.9*	*2.2*	*3.7*							*−3.2*	*5.9*	*8.4*

Note

Bias, intra‐day precision (RSD_R_), inter‐day precision (RSD_T_), and recovery efficiency (RE) are given in percent (%).

### 2.4 Sample preparation

Cal, QC, and sample preparation was performed on ice. As negative control, Li‐heparin tubes were filled with PBS (10 mL), vortexed for 10 seconds, and incubated for 10 minutes. For analysis, 400 µL, Cal, QC, or authentic samples were mixed with 10 µL internal standard mix and 750 µL 2 mol/L NaOH/0.5 mol/L KCl in a 20 mL GC headspace vial and sealed directly. Samples were vortexed for 5 seconds and set on the bench to reach room temperature. Afterward samples were analyzed as described above. Choline and carnitine solutions were prepared in PBS (5 mmol/L) and analyzed accordingly.

### 2.5 Method development

In the following, QC Med was used for evaluating different HS conditions. Incubation time was investigated in 10 minutes steps from 10 to 30 minutes. Incubation temperature was tested at 60, 70, and 80°C. For liberation of the free amine, NaOH (0.5‐2.0 mol/L) and KCl (0.3‐0.5 mol/L) were used. Different sample volumes were evaluated (50 µL, 100 µL, 250 µL, and 400 µL).

### 2.6 Method validation

Six replicates (on six different days) at each concentration level were analyzed according to the aforementioned procedure. The regression lines were calculated using, a quadratic weighted [1/*x* ^2^] least‐squares regression model. Daily regression lines were used to back‐calculate the concentration of each calibrant. The back‐calculated concentrations of all calibration samples were compared to their corresponding theoretical values. Quantitative accuracy was limited to be within 20% of target. Isopropylamine was applied as internal standard for all target analytes. QC samples (Low, Med, High) were prepared and analyzed in duplicate on each of 8 days. Accuracy was determined in terms of bias as the percent deviation of the mean calculated concentration at each QC level from their respective nominal concentration. Intra‐day and inter‐day precisions were calculated as relative standard deviation (RSD) according to Peters and coworkers.^30^ Recovery (RE) was investigated at QC Med level using six different urine and blood sources according to the simplified approach described by different research groups.^27^, ^31^ Different storage conditions (room temperature, 4°C and −20°C) were investigated at QC Med level. Furthermore, QC Med was submitted to five freeze‐thaw cycles. After each cycle, samples were analyzed according to the presented procedure. Between each freeze‐thaw cycles, samples were kept for at least 24 hours in the freezer. For LoQ, predefined goals for bias (<15%) and RSD (<15%) at QC low level were set.^32^

### 2.7 Applicability

Blood samples were obtained from 11 different and apparently healthy volunteers of the Kantonsspital Aarau who provided written informed consent in accordance with the Declaration of Helsinki. All blood and urine samples were collected after an overnight fastening period (at least 4 hours). Blood plasma was obtained after immediate centrifugation at 3000 *g* for 5 minutes. Supernatant was immediately separated and stored at −20°C until further usage. Urine was directly acidified by dropwise addition of hydrochloric acid solution until pH was below 2. Dilution factor from acidification was negligible for all samples (approx. 20 µL of 25% HCl in 10 mL urine).

### 2.8 Data analysis

Thermo Excalibur software (2.2 SP1.48) was used for peak integration and quantification of data. GraphPad Prism 7 (GraphPad Software) was used for statistical analysis and illustrations.

## 3 RESULTS

### 3.1 Method development

**Figure 1:**
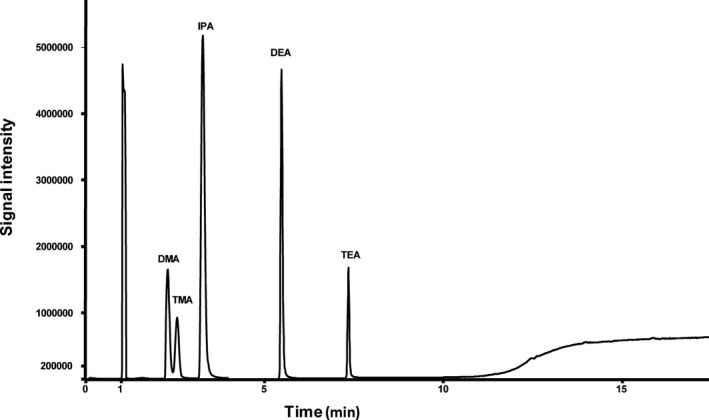
Total ion chromatogram of target analytes and internal standard

**Figure 2:**
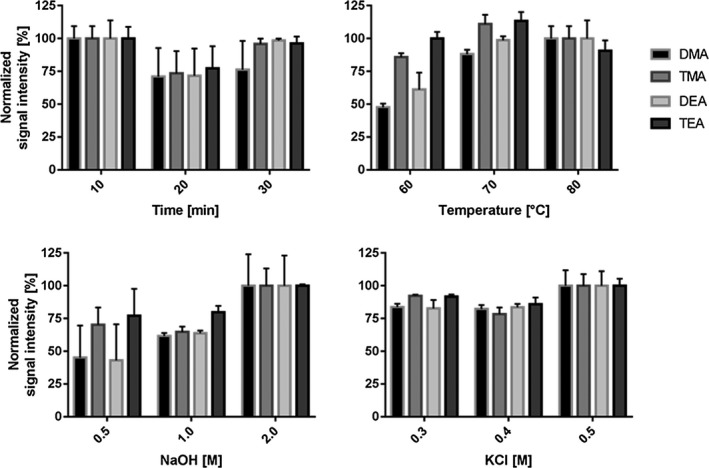
Optimization of single conditions for static HS‐GC. All experiments were performed with QC Med in triplicate (n = 3). Data were normalized to the highest obtained values

**Figure 3:**
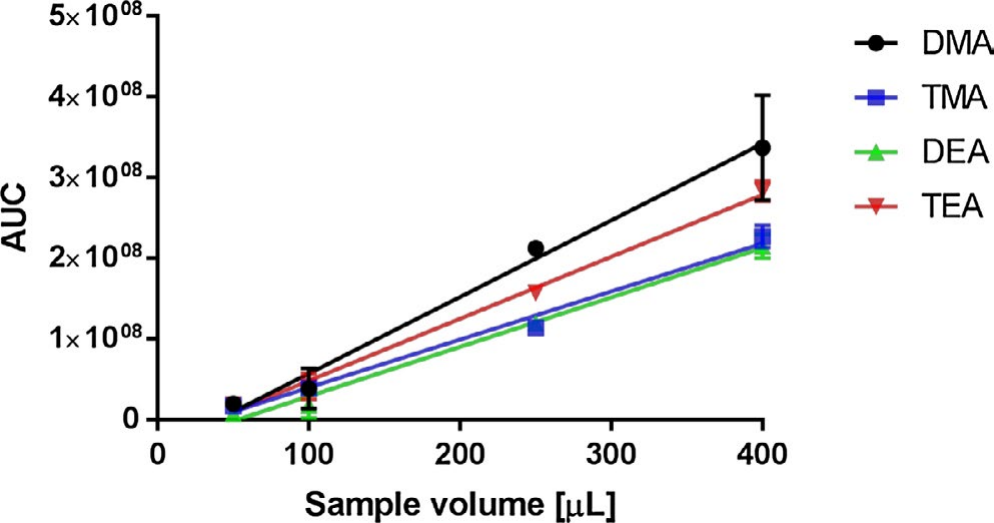
Linearity experiments were performed in triplicate using QC Med samples (n = 3) using different sample volumes

Chromatographic separation of DMA, TMA, DEA and TEA was performed on a Restek volatile amine column. Baseline separation of all analytes could be achieved after 7.5 minutes (Figure 1). Several different flows (data not shown) were tested, and finally, a flow of 2 mL/min helium was applied for all further analysis. In the following, QC Med was used for evaluating different preanalytical conditions. In each experimental setup, highest signal intensities were set to 100%. Elevated NaOH and KCl concentrations lead to increased signal intensities of all target analytes (Figure 2). However, further increase of the volume of NaOH/KCl solution decreased signal intensity again (Figure S1). Additionally, incubation time and temperature play a crucial role in HS‐GC and were also investigated in detail. Highest signal intensity was observed with 10 minutes of incubation at a temperature of 70°C (Figure 2). Multiple injections from the same vial lead to a decrease in signal intensity (Figure S2). Also, different sample volumes were systematically investigated (50 µL, 100 µL, 250 µL, and 400 µL). As shown in Figure 3, there was a linear increase in signal intensity observed. Finally, following parameters were set and used for method validation: injector temperature: 200°C; incubation time: 10 minutes; incubation temperature: 70°C; 750 µL NaOH 2 mol/L /KCl 0.5 mol/L; sample volume: 400 µL; split ratio: 7.

### 3.2 Method validation

Initially, TMA_d9_ was used as internal standard. Since we observed a high inter‐assay variance of signal intensity, IPA replaced TMA_d9_ as internal standard for the method validation and all further assays. Isopropylamine could clearly be separated from all other SCVA. The use of internal standards corrected for any apparent loss of analytes during the liberation, static headspace extraction, and split injection. Calibration curves using six concentration levels with six replicates each were constructed to evaluate the calibration model. Calibration ranges for all analytes are given in Table 1. Accuracy was given in terms of bias as the percent of deviation of the mean calculated concentration compared to the theoretical value. No carryover was detected in blank samples injected after Cal 1 and QC High. Intra‐day and inter‐day precisions were below 7% for all SCVA at the higher calibration range. At the lower end of the calibration curve, RSDs for TMA and TEA were below 9%. Only DMA and DEA showed a higher RSD of around 12%. After 2 weeks at room temperature or at 4°C, DMA and DEA concentration was reduced to around 80% of the starting concentration, whereas TMA and TEA concentrations decrease to around 60% and 70%, respectively. After multiple freeze‐thaw (FT) cycles, concentration of all target analytes was decreased. FT cycles were performed over 5 days, and relative concentration of 90%, 86%, 83%, and 79% for DMA, TMA, DEA, and TEA was detected, respectively. Recovery data (RE) were calculated from six different Li‐heparin plasma and urine sources and listed in Table 2. All analytes could be extracted with REs over 75% from both matrices. Overall, the REs were highly reproducible with RSDs less than 7%. Only for DMA in plasma and urine RE were 75% and 112%, respectively (Table 1).

**Table 2:** Metabolite concentrations in plasma and urine samples of 11 apparently healthy volunteers

	Matrix	PBS	1	2	3	4	5	6	8	9	10	11
DMA [µmol/L]	Urine		434.8	672.2	130.7	268.2	181.8	469.3	306.3	429.5	385.1	160.7
	Plasma	nd	nd	nd	nd	nd	nd	nd	nd	nd	nd	nd
TMA [µmol/L]	Urine		2.5	3.1	1.1	1.8	2.1	3.5	2.3	2.2	85.3	1.2
	Plasma	nd	4.9	4.6	5.3	4.6	4.5	4.5	4.7	6.3	5.3	6.2

Abbreviation: nd, not detected.

### 3.3 Applicability

**Figure 4:**
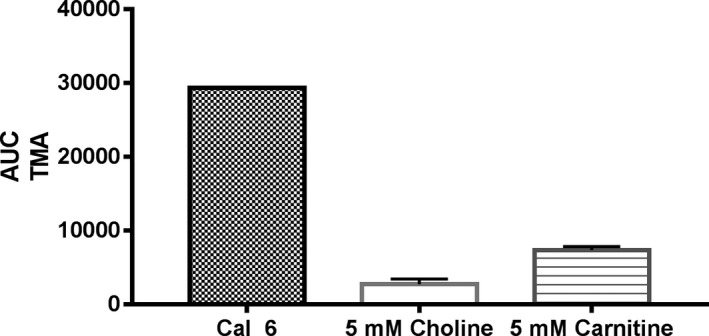
AUC of TMA detected in Cal 6 is compared to AUC of TMA found in choline and carnitine samples (5 mmol/L) under forced alkaline and heated conditions (n = 3), respectively

The developed method was successfully applied for selected SCVA determination in human plasma and urine from 11 healthy volunteers. Samples of the same individual were analyzed in the same run. As shown in Table 2, no target analytes were detected in negative controls. In plasma, only TMA was found. The concentration range of TMA was between 4.5 and 6.3 µmol/L. In urine, TMA and DMA were present. The amount of TMA and DMA found in the analyzed urine samples was between 1.1 to 3.1 µmol/L and 130 to 673 µmol/L, respectively. Only one urine sample showed approx. 25 times higher TMA concentration (85.3 µmol/L). Additionally, single carnitine and choline solutions were analyzed according to the presented method. TMA was detected in both solutions. However, compared to the Cal 6, applied HS conditions resulted in three to five times lower signal intensities of TMA for carnitine and choline, respectively (Figure 4). DMA was not present in urine and plasma samples. Only in one urine sample TEA was detected in a concentration of 0.5 µmol/L.

## 4 DISCUSSION

For short‐chain fatty acids and for SCVA, polyethylene glycol based columns were broadly used for separation. After intense literature search, we decided to focus on a newly developed volatile amine column.^13^, ^33^, ^34^ The stationary phase is highly suitable for polar and basic target analytes. Although direct injection of aqueous samples is feasible with this column, we applied static headspace chromatography to analyze SCVA in biological samples like plasma and urine to reduce possible contamination with non‐volatile substances. Since target analytes and their fragmentation pattern are closely related, baseline separation was required. Identification was done by the retention time of reference compounds. Based on their basicity, SCVA are trapped as ion‐pairs in acid solutions and vaporize poorly. Analysis of up to 1000 µmol/L TMA*HCl in H_2_O only revealed small peaks in static HS‐GC (data not shown). For liberation of the free amine, different concentrations of NaOH were investigated in detail. Increase of the polarity of the aqueous phase by adding high KCl concentrations further decrease the solubility of volatile organic compounds in the aqueous matrix and promote their transfer into the HS.^35^ Since increasing the volume of liberation solution decreased signal intensities of all target analytes were observed, we assume that higher content of water traps the polar analytes in the aqueous phase due to equilibrium issues between the solution and the headspace. To prevent condensation in the HS syringe, the temperature of the syringe was always higher by 10°C in comparison with the incubation temperature. As shown in Figure 3, signal intensity was linear in relation to sample volume. In respect of the LoQ's and the expected concentrations in human urine and blood samples, all further experiments were performed using 400 µL of sample. For calibration, different models were tested. For all target analytes, no linear relationship between concentration and signal intensity could be detected across the calibration range. Based on lowest variances in back‐calculated concentrations of standard solutions, a 1/*x* ^2^ weighted quadratic calibration model was used to account for unequal variances (heteroscedasticity). The calibration range for all target analytes is shown in Table 1. A slight curvature was indicated by the quadratic regression model indicated for all target analytes. Predefined criteria for LoQ were met at QC Low levels for all target analytes. At QC Low level, only DMA and DEA showed a higher RSD of approx. around 12%. According to international guidelines for bioanalytical methods, this is still within an acceptable range.^29^ Since all target analytes are highly volatile, decreasing concentrations under different storage conditions and several freeze‐thaw cycles were observed. Therefore, we recommend preparing standards and QC solutions freshly.

Based on contact to experts in the field, we aim to measure the following markers in upcoming clinical studies to evaluate the predictive values of DMA and TMA under different metabolic disorders. In urine, DMA and TMA were detected and correspond to previously published values of a healthy population.^27^, ^36^ For all plasma samples, no DMA was detected which is also in line with common knowledge. Interestingly, we found five times higher TMA concentrations in plasma as reported by Bain et al^37^ but two times lower values than Zeisel et al.^38^ As described earlier, it is likely that DMA is present as a contaminant in EDTA plasma tubes.^26^ To exclude production process related contamination with SCVA, Li‐heparin tubes were filled with 10 mL PBS as negative control. No contamination with SCVA was detected. We decided to use Li‐heparin tubes without separator gel for blood sampling since gel separator tubes may affect analytical results.^39^ As reported by Bain et al,^40^ quaternary ammonia compounds can undergo a Hofmann‐elimination resulting in ex vivo generation of TMA. Therefore, carnitine and choline were prepared in PBS (5 mmol/L each) and analyzed according to the described method. Although the applied concentration is up to 100‐fold higher as reported for plasma,^5^ only a slight TMA signal was detected under current HS conditions (Figure 4). One may speculate if incubation temperature of 50°C and missing salting out conditions—as reported by Bain and coworkers—are not sufficient to equilibrate the HS. Therefore, plasma TMA values, reported in here, are higher compared to those reported previously.^40^ As expected, no DEA was found in urine or plasma, respectively. Interestingly, in only one urine sample TEA was calculated to 0.5 µmol/L. Identity was confirmed by retention time and fragmentation pattern (Figure S4). The occurrence of TEA will be inspected in detail but is not further discussed in here.

In conclusion, the presented analytical method allows the simultaneous accurate and precise quantification of four pharmacologically and toxicologically important short‐chain volatile amines in Li‐heparin plasma and urine. The lack of derivatization further accelerates the preanalytical phase and avoids errors in sample preparation, respectively. The method met the validation criteria for all analytes. Subsequently, we demonstrated its practicability by the successful analysis of 11 paired Li‐heparin plasma and urine samples of healthy volunteers. We aim to investigate SCVA in further clinical and toxicological studies. The results of these studies will be presented elsewhere.

## AUTHOR CONTRIBUTIONS

This study was designed by CS. CS, MDA, and PN equally performed the experimental work. LB and PN collected serum and urine samples. JAF set up the chromatographic conditions. The manuscript was written through contributions of all authors. All authors have given approval to the final version of the manuscript.

## Data Availability

Research data have been provided in the manuscript and supporting information.
