# Supplementary material for: Derivatization‐free determination of short‐chain volatile amines in human plasma and urine by headspace gas chromatography‐mass spectrometry
Source: J Clin Lab Anal. 2019 Oct 8;34(2):e23062. doi: 10.1002/jcla.23062 (PMC7031570; doi:10.1002/jcla.23062)
Supplement: Supplementary file 1 [file JCLA-34-e23062-s001.docx]

Supporting Information

**Derivatization-free determination of short chain volatile amines in human plasma and urine by Headspace Gas Chromatography Mass Spectrometry**

Peter Neyer^a^, Luca Bernasconi^a^, Jens A. Fuchs^b^, Martina D. Allenspach^b^, Christian Steuer^b^*

^a^Kantonsspital Aarau, Institute of Laboratory Medicine, Tellstrasse 1, CH-5001 Aarau, Switzerland

^b^ETH Zurich, Institute of Pharmaceutical Sciences, Vladimir-Prelog-Weg1-5/10, CH-8093 Zurich, Switzerland

*Corresponding author:

Tel.:+41 628385315; Fax: +41 628385399

E-mail address: [christian.steuer@pharma.ethz.ch](mailto:christian.steuer@pharma.ethz.ch)

**Contents Page**

**Table S1** Temperature gradient **2**

**Figure S1** Different volumes of 2 M NaOH/ 0.5 M KCL-solution **3**

**Figure S2** Iterative injections from the same vial **3**

**Figure S3** Different split ratios tested **4**

**Figure S4:** Extracted ion chromatogram for mass 101 and corresponding **4**

fragmentation pattern

Table S1: Temperature gradient

| Step | Temperature [°C] | Increase [°C/min] | Time [min] | Flow [ml/min] |
| --- | --- | --- | --- | --- |
| 1 | 40 | Hold | 5 | 2 |
| 2 | 200 | 25 | 115 | 2 |
| 3 | 200 | Hold | 5 | 2 |
| 4 | 250 | 10 | 3 | 2 |
| 5 | 250 | Hold | 3 | 2 |


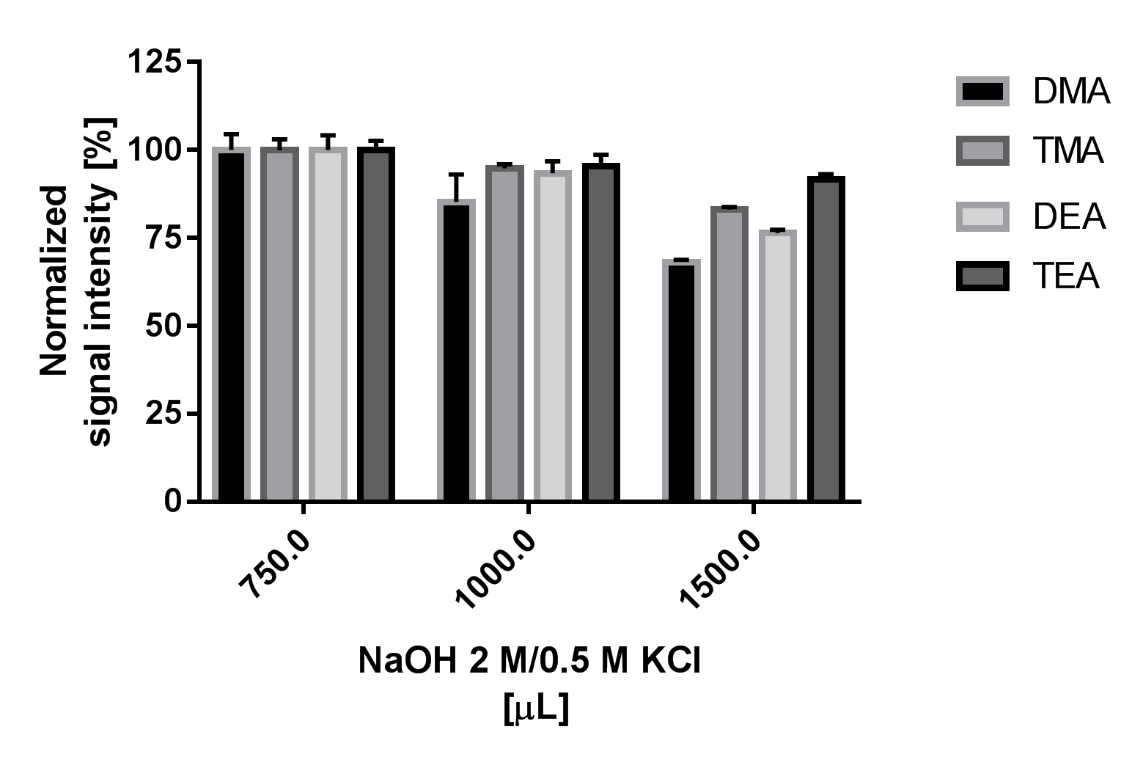


Figure S1: Different volumes of 2 M NAOH/ 0.5 M KCl-solution
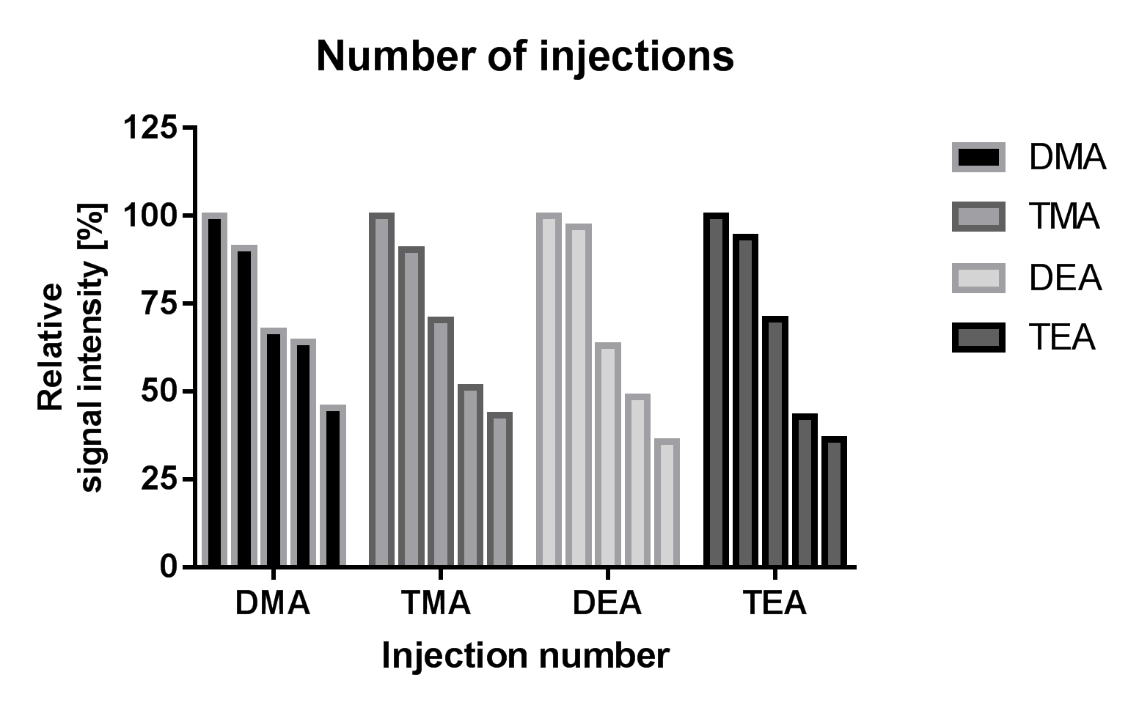


Figure S2: Iterative injections from the same vial.


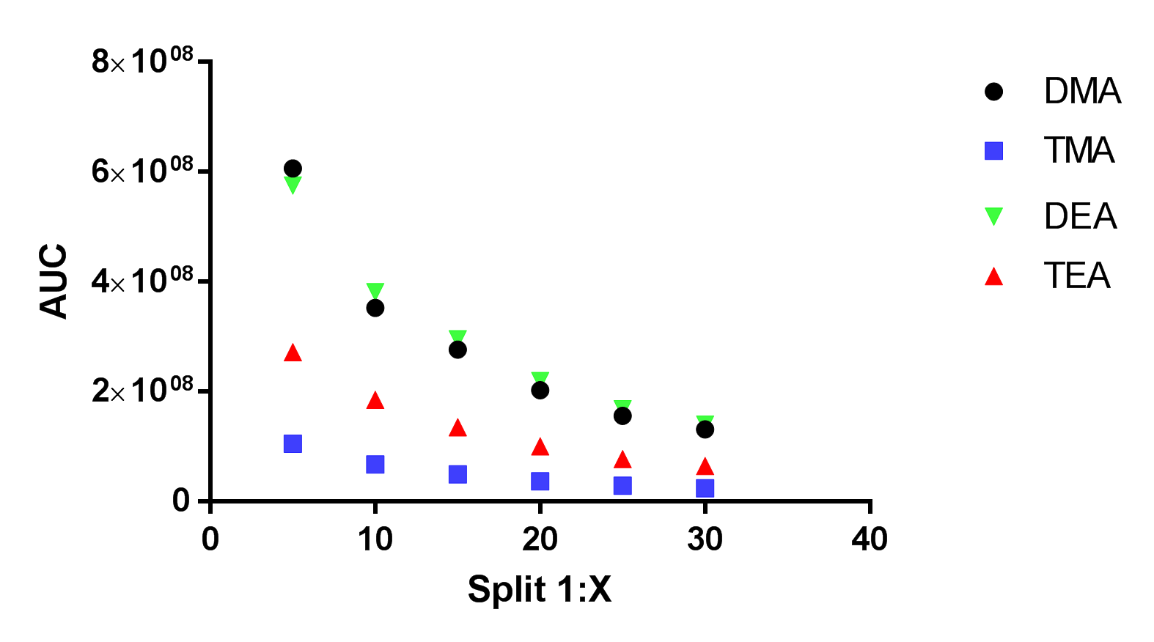


Figure S3: Different split ratios tested.


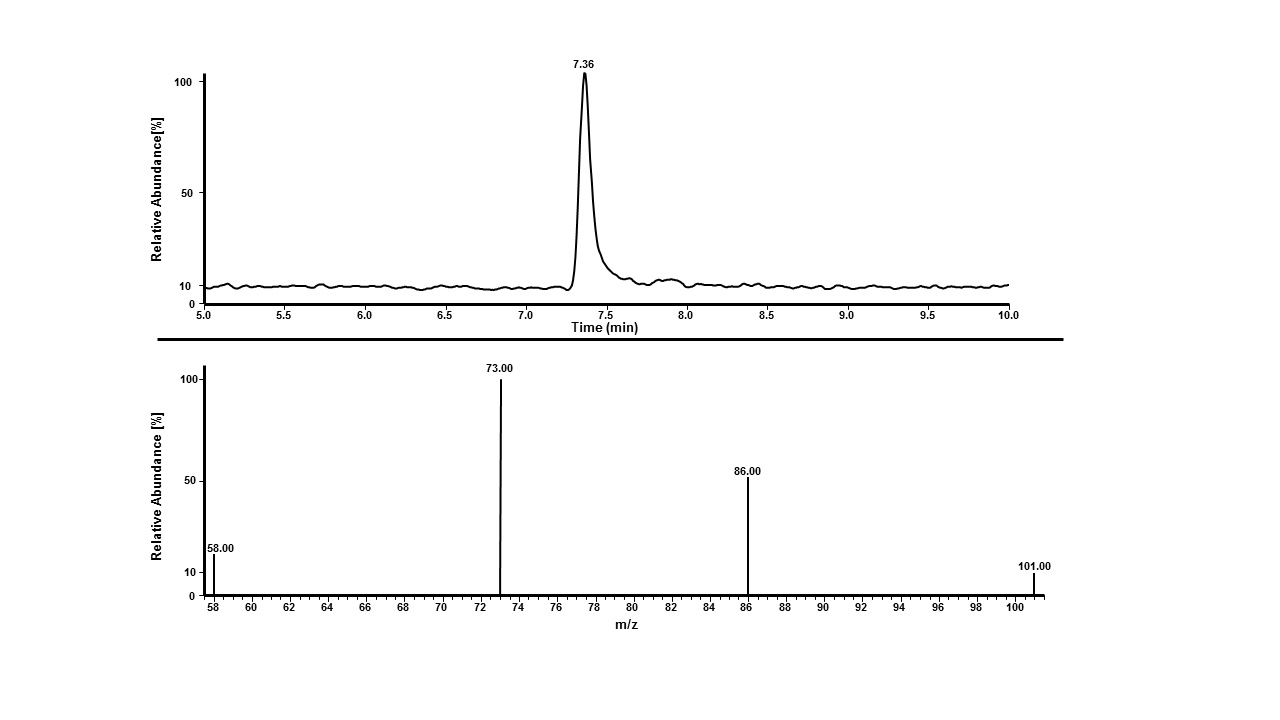


Figure S4: Chromatogram of urine sample 6 and corresponding fragmentation pattern of peak eluting at 7.36 min
